# Control Architecture for Human-Like Motion With Applications to Articulated Soft Robots

**DOI:** 10.3389/frobt.2020.00117

**Published:** 2020-09-11

**Authors:** Franco Angelini, Cosimo Della Santina, Manolo Garabini, Matteo Bianchi, Antonio Bicchi

**Affiliations:** ^1^Centro di Ricerca “Enrico Piaggio”, Università di Pisa, Pisa, Italy; ^2^Soft Robotics for Human Cooperation and Rehabilitation, Fondazione Istituto Italiano di Tecnologia, Genoa, Italy; ^3^Dipartimento di Ingegneria dell'Informazione, Università di Pisa, Pisa, Italy; ^4^Robotic Mechatronic Center, German Aerospace Center (DLR), Institute of Robotics and Mechatronics, Cologne, Germany; ^5^Department of Informatics, Technical University Munich, Munich, Germany; ^6^Cognitive Robotics Department, Delft University of Technology, Delft, Netherlands

**Keywords:** motion control algorithm, motor control, natural machine motion, articulated soft robots, human-inspired control, compliant actuation

## Abstract

Human beings can achieve a high level of motor performance that is still unmatched in robotic systems. These capabilities can be ascribed to two main enabling factors: (i) the physical proprieties of human musculoskeletal system, and (ii) the effectiveness of the control operated by the central nervous system. Regarding point (i), the introduction of compliant elements in the robotic structure can be regarded as an attempt to bridge the gap between the animal body and the robot one. Soft articulated robots aim at replicating the musculoskeletal characteristics of vertebrates. Yet, substantial advancements are still needed under a control point of view, to fully exploit the new possibilities provided by soft robotic bodies. This paper introduces a control framework that ensures natural movements in articulated soft robots, implementing specific functionalities of the human central nervous system, i.e., learning by repetition, after-effect on known and unknown trajectories, anticipatory behavior, its reactive re-planning, and state covariation in precise task execution. The control architecture we propose has a hierarchical structure composed of two levels. The low level deals with dynamic inversion and focuses on trajectory tracking problems. The high level manages the degree of freedom redundancy, and it allows to control the system through a reduced set of variables. The building blocks of this novel control architecture are well-rooted in the control theory, which can furnish an established vocabulary to describe the functional mechanisms underlying the motor control system. The proposed control architecture is validated through simulations and experiments on a bio-mimetic articulated soft robot.

## 1. Introduction

Daily activities of human beings are a clear example of the exceptional versatility of their motor control system. Tasks that are still challenging for robots are indeed easily executed by people. Responsible for such a high level of performance are the musculoskeletal system and the Central Nervous System (CNS). The musculoskeletal system allows to exert forces and to percept the external world through a multitude of receptors. One of the main characteristics of this system is its compliant nature. Indeed, body flexibility provided by muscles and tendons enables features like energy efficiency, power amplification and shock absorption (Roberts and Azizi, 2011).

The same feature are usually hard to be achieved by traditional rigid robots. Inspired by the effectiveness of the biological example, researchers developed robots with compliant elements to mimic the animal body. This novel generation of systems, namely soft robots, can be categorized as invertebrate-inspired or vertebrate-inspired (Della Santina et al., 2020). The latter class includes *articulated soft robots*, which are systems with rigid links and elasticity lumped at the joints (Albu-Schaffer et al., 2008). In this paper, we focus on the latter category, i.e., robots actuated by series elastic actuators (SEA) (Pratt and Williamson, 1995) or variable stiffness actuators (VSA) (Vanderborght et al., 2013). The musculoskeletal system of vertebrates allows to adjust its dynamics, for instance, it allows to vary joint stiffness via co-contraction of antagonistic muscles. Agonistic-antagonist VSAs mimic this mechanism as described in Garabini et al. (2017), thus they try to replicate the working principle of the human musculoskeletal system.

Several works in literature describe how the features of a flexible body can be conferred also to a robot through different solutions (Landkammer et al., 2016; Zhang et al., 2019; Pfeil et al., 2020). Particularly relevant are the solutions that completely replicate the whole structure of the human musculoskeletal system. For examples, Kenshiro (Asano et al., 2016) is a humanoid robot reproducing the human skeleton and muscle arrangement. Marques et al. (2010) presents ECCE, an anthropomimetic humanoid upper torso. Jäntsch et al. (2013) proposes Anthrob, a robot mimicking a human upper limb.

Yet, controlling soft robots still remains a very challenging task. The reason is that articulated soft robots have highly non-linear dynamics, presenting also hysteresis, bandwidth limitation and delays. Therefore, obtaining an accurate and reliable dynamic model is not a trivial task that could directly affect the performance of model-based control techniques. Moreover, articulates soft robots present anatomical degrees of freedom (DoFs) redundancy, because they typically have more than one motor per joint, and they may have kinematic DoFs redundancy, depending on the platform. The majority of existing model-based control approaches has the strong drawback of requiring an accurate model identification process, which is hard to be accomplished and time-consuming. In Buondonno and De Luca (2016) feedback linearization of VSA is faced. In Zhakatayev et al. (2017) an optimization framework to minimize time performance is proposed. In Keppler et al. (2018) the Authors propose a controller to achieve motion tracking while preserving the elastic structure of the system and reducing the link oscillations. On the other hand, model-free algorithms are promising, but usually require long-lasting learning procedures and face generality issues (Angelini et al., 2018; Hofer et al., 2019).

However, the complexity of the articulated soft robot body is analogous to that of their source of inspiration. Indeed, the human body is a complex system that presents an unknown non-linear dynamics and redundancy of degrees of freedom (DoFs). Despite that, the CNS is able to cope with these issues, fully exploiting the potential of the musculoskeletal system. For this reason, in this work, we analyze the effectiveness of a bio-inspired algorithm to control bio-mimetic robots.

To the authors best knowledge, despite the variety of approaches in the motor control field, an architecture based on control theory able to present at the same time various CNS behavior is still lacking for articulated soft robots (Cao et al., 2018; Ansari et al., 2019). The study of the human CNS has been already exploited to enhance robot capability. For instance, in Medina et al. (2019) the Authors propose a method for modeling human motor behavior in physical and non-physical human-robot interactions. Based on previous observations, the developed model is able to predict the force exerted during the interaction. Capolei et al. (2019) presents a cerebellar-inspired controller for humanoid robot moving in unstructured environment. The controller is based on machine learning, artificial neural network, and computational neuroscience. In Kuppuswamy et al. (2012) the Authors propose a motor primitive inspired architecture for redundant and compliant robots. Lee et al. (2018) proposes a model of human balancing with the goal of designing a controller for exoskeleton.

In this work, our goal is to make a step further toward the development of human-inspired controllers for articulated soft robots: taking inspiration from motor control theories, we implemented a hierarchical control architecture exhibiting well-known characteristics of human motor control system (i.e., learning by repetition, anticipatory behavior, synergistic behavior). Such a control framework is a proper combination of feedback control, feedforward, Iterative Learning Control, and Model Predictive Control. The goal is to design a bio-mimetic control architecture for bio-inspired robots, focusing on trajectory planning and tracking tasks.

A major contribution of this work is to show how well-established paradigms belonging to the control theory can be used to approach the motor control problem. Finally, the authors want to clearly state that is beyond the scope of this work to infer possible neurophysiological implications based on the presented control framework.

Our belief is that a control system able to work like the CNS, such the one proposed here, can successfully manage a soft robotic system. We test here this hypothesis, among with the human-like behaviors, both in simulation and in experiments, using as testbed robots actuated by VSAs.

## 2. The Biological Inspiration

The unparalleled performance of the animal CNS are an ambitious goal for the robotic community, especially because the issues faced by the CNS are very similar to the ones occurring in robots, i.e., unknown non-linear dynamics and redundancy of degrees of freedom. These are (Latash, 2012):

- *Unknown non-linear dynamics*. The human body is a complex system, with strong non-linearities at every level. Moreover, environmental force fields can not be known *a priori*.
- Degree of freedom (DoF) redundancy. The human body presents three types of redundancy. *Anatomical*—human body is characterized by a complex highly redundant structure. The number of joints is greater than the number of DoFs necessary to accomplish a generic task, and the number of muscles is greater than the number of joints. *Kinematic*—infinite joints trajectories can achieve the same task, or simply perform the same end effector point to point movement. *Neurophysiological*—each muscle consists of hundreds of motor units, and they are activated by moto-neurons that can spike with different frequency (hundreds of variables).

For this reason, we use the motor control theory as a source of inspiration for our controller.

### 2.1. Hierarchical Nature of the Central Nervous System

There are several evidences that the Central Nervous System can cope with the incredible complexity of the musculoskeletal apparatus by relying on a hierarchical organization of subsequent simplifications of the control problem (Swanson, 2012; Hordacre and McCambridge, 2018). For example, the Bernstein classification (Bemstein, 1967) categorizes the construction of movement in six levels, from symbolic reasoning to muscle tone activation. Level A is called *rubro-spinal level* or *paleokinetic level*, and it provides reflex function and manages muscle tone. Level B, i.e., *thalamo-pallidal level*, is the level of synergies and patterns and produces coordinate movement patterns. Finally, level C1, is the *striatal* or *extrapyramidal level*. This is one of the two levels of the *spatial field* level, and it specifies a way to reach performance defined by higher levels. The other three levels, C2, D, and E, describe higher level of abstractions, as meaningful actions and information transmission. Therefore, they will not be treated in by the proposed control architecture.

### 2.2. Some Salient Characteristics of the Human Motor Control

**Figure 1:**
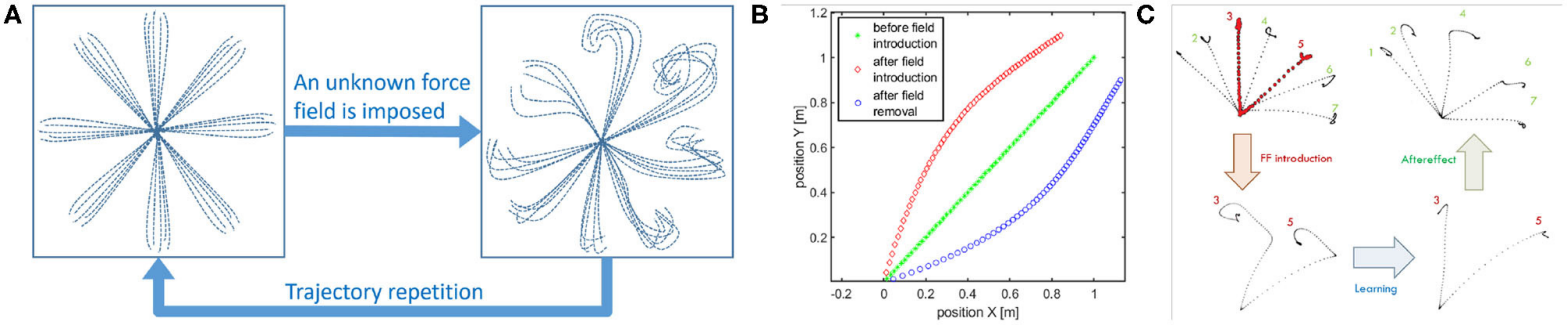
Representation of some human behaviors considered in this work. Learning by repetition **(A)**: a subject is able to reach a series of point in space with its end effector, when a force field is imposed the trajectories result deformed, repeating the reaching trials many times the subject results able to restore the initial behavior. Aftereffect in known trajectories: **(B)** Hand trajectories of a typical point to point movement. The typical movement is a strict line. If a force field is introduced the trajectory is firstly deformed. After some repetitions the strict movement is recovered. If the force field is then removed the hand trajectory is deformed in a way specular to the first deformation. This is called aftereffect. Aftereffect in unknown trajectories: **(C)** Hand trajectories of typical point to point movements. When the force field is introduced the subject make experience through learning by repetition of just trajectories 3 and 5. When the force field is removed aftereffect is present on trajectories not experienced closer to trajectories 3 and 5: trajectory 4 presents maximum aftereffect, trajectories 1 and 7 presents negligible aftereffect (image obtained from an elaboration of images in Gandolfo et al., 1996).

**Figure 2:**
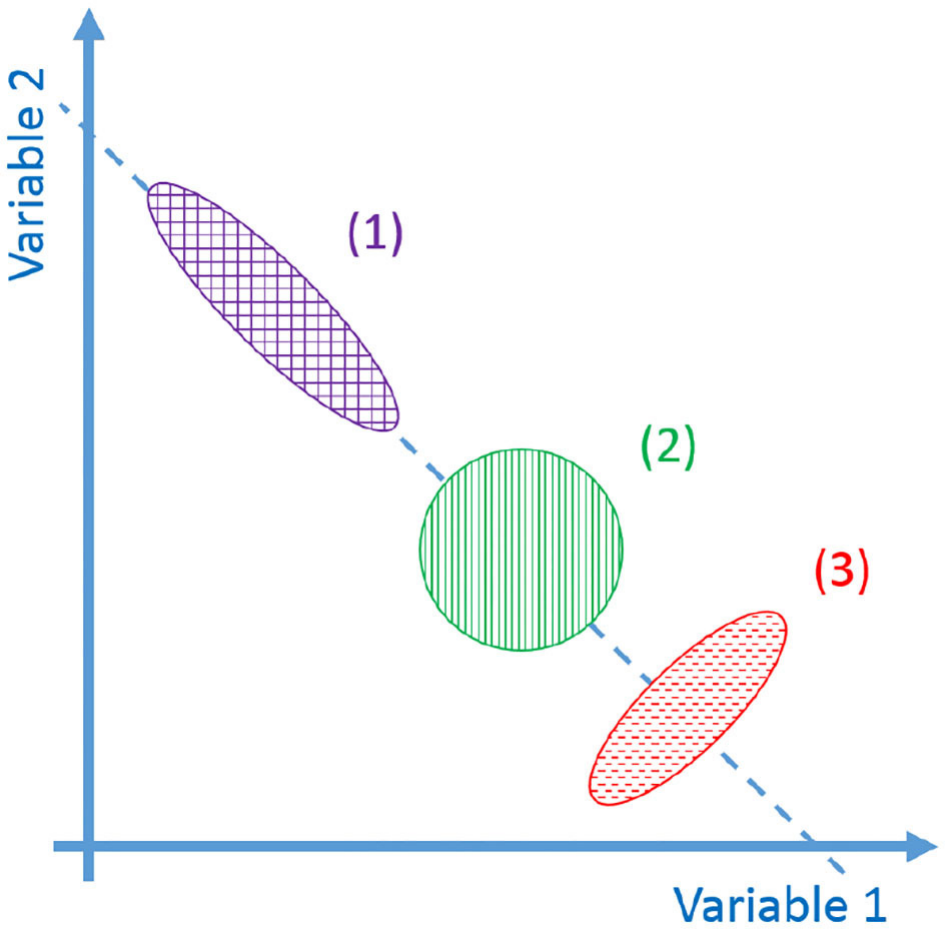
Representations of the synergistic behavior. In the figure there are different possible distributions of task configuration in task space. The dashed line is locus of configurations that meets the task. *V*_good_ is the variance of the distribution along the dashed line, *V*_bad_ is the variance in the orthogonal directions. The fact that *V*_good_ > *V*_bad_ indicates that a task synergy exists (1). If *V*_good_ ≃ *V*_bad_ no synergy exists (2). If *V*_good_ << *V*_bad_ a destabilizing synergy exists (3).

In this section we list a few of salient characteristics of the neural control architecture that we consider of paramount importance for the human motion performance, and that we aim at replicating on the considered bio-mimetic robots. In the remainder of the article we will often refer to them as (i)–(v). These peculiar characteristics of the CNS are:

- Learning by repetition (Shadmehr and Mussa-Ivaldi, 1994): CNS inverts an unknown dynamic over a trajectory, repeating it several times. Figure 1A represents a classical experiment. It is possible to notice that the subject is asked to reach some points in the workspace. Then a force field is introduced. Initially, trajectories are strongly deformed. After repetitions of the same movements, performances obtained before the introduction of the force field are achieved again. The same behavior can be found in the development, where the CNS needs to adapt to its own dynamics.
- Anticipatory behavior (Hoffmann, 2003): ability of CNS to usually anticipate the necessary control action relying on sensory-motor memory. The acquired previous experiences cause a shift in the control action from closed loop to open loop. Anticipatory behavior is fundamental in many human activities, such as manipulation (Fu et al., 2010), coordinated (Flanagan and Wing, 1993), and fast movements (Haith et al., 1988).
- Aftereffect over a learned trajectory (Lackner and Dizio, 1998) and aftereffect over unknown trajectories (Gandolfo et al., 1996). After recovering the performance loss due to the introduction of the external force field, by removing the force field, subjects exhibit deformations of the trajectory specular to the initial deformation due to the force field introduction. This behavior is called mirror-image aftereffect Figure 1B. This effect arises also in novel trajectories as depicted in Figure 1C.
- Synergistic behavior (Latash, 2010): synergy can be defined as “*[…] a hypothetical neural mechanism that ensures task-specific co-variation of elemental variables providing for desired stability properties of an important output (performance) variable*.” Given an “important output variable” we can define two variables *V*_good_ and *V*_bad_. *V*_good_ is the variance through the directions where output is constant and the constraints are verified (named *uncontrolled manifold*), while *V*_bad_ is the variance in the other directions (Scholz and Schöner, 1999). The system presents a synergistic behavior when *V*_good_ > *V*_bad_. Figure 2 visually explains this point.
- Re-plan of anticipatory action: CNS modifies the anticipatory motor actions on-line if the goal changes (e.g., Soechting and Lacquaniti, 1983), or if the sensory outcome is different from the expected one (e.g., Engel et al., 1997). Note that this is fundamentally different from feedback. Indeed, feedback actions are proportional to the instantaneous error, while re-plan of anticipatory action depends on the outcome of the task.

## 3. Problem Statement

Inspired by the biological example, we design the control architecture with a hierarchic structure similar to the one of CNS. In particular we reproduce the first three levels of the Bernstein classification (Bemstein, 1967) (briefly summarized in section 2.1) with the goal of executing a task reference ν generated by the three higher abstraction levels. Furthermore, the controller has to reproduce the peculiar behaviors of the human CNS described in section 2.2.

**Figure 3:**
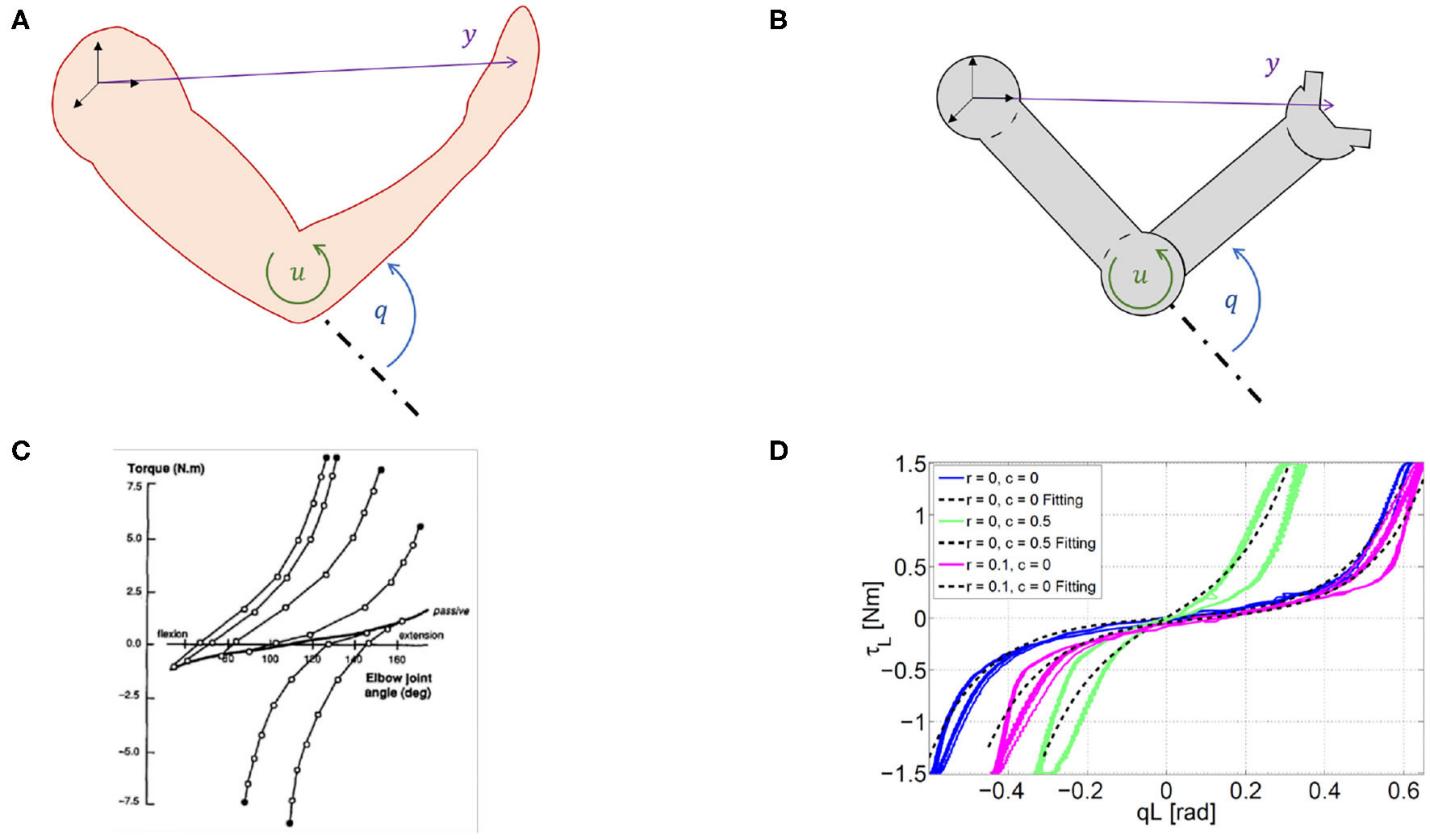
Similarity between humans and robots. Variable definitions in humans **(A)** and robots **(B)**. *q* ∈ ℝ^*n*^ are the Lagrangian variables, $x={\left[q^{\text{T}},{\overset{∙}{q}}^{\text{T}}\right]}^{\text{T}}\in {\mathbb{R}}^{2n}$ is the state vector, *u* ∈ ℝ^*m*^ is the input and *y* ∈ ℝ^*l*^ is the output. These variables are valid both for biological systems and articulated soft robots. Experimentally measured force–length characteristics in natural **(C)** and robotic **(D)** system. **(C)** Elastic characteristic of agonist and antagonist muscles acting on the elbow joint in the human, taken from Gribble et al. (1998). **(D)** Elastic characteristic of a agonist and antagonist variable stiffness actuator (Garabini et al., 2017).

**Figure 4:**
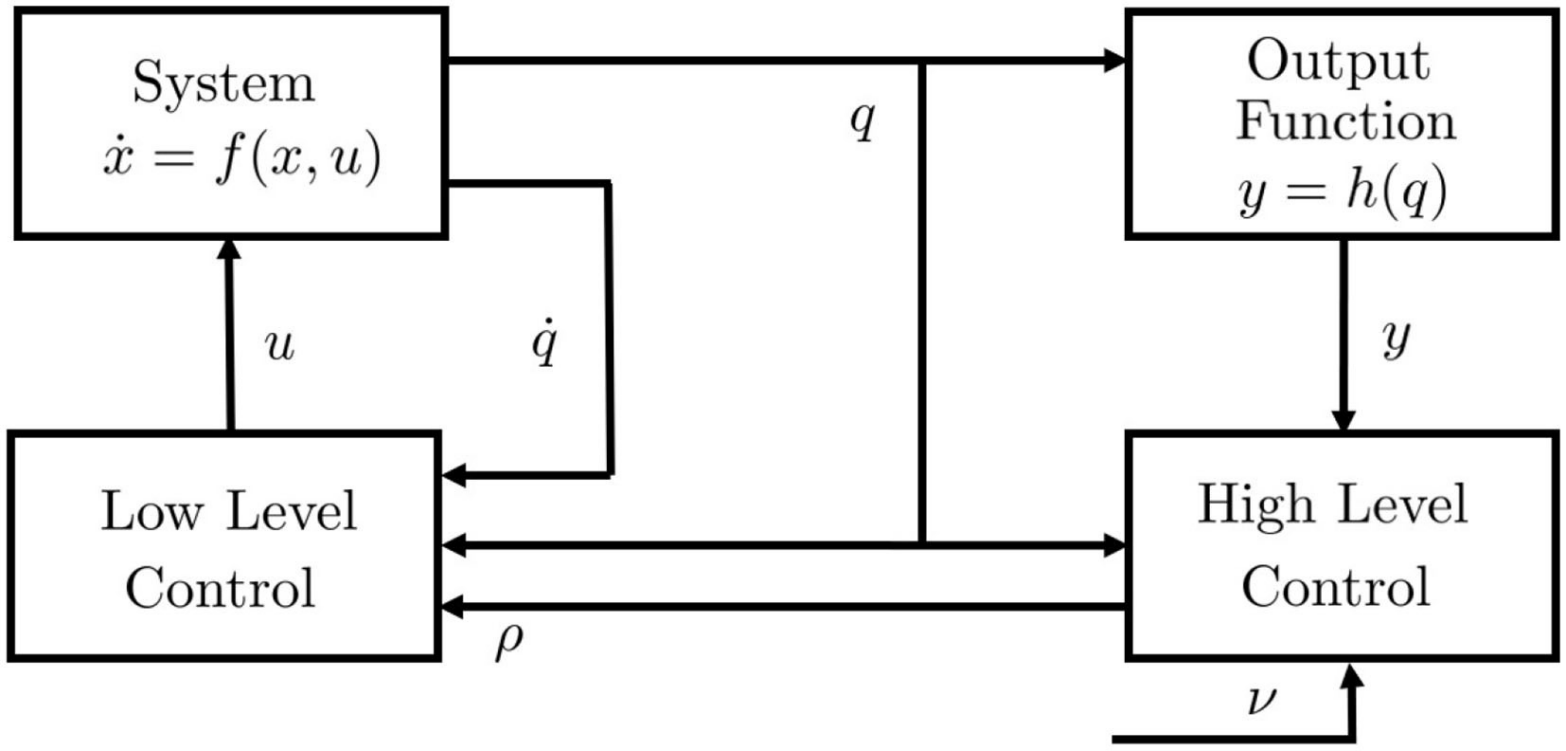
Control structure. *u* is the low level control variable or efferent action, ρ is the high level control variable, ν is the reference in the task space, *q* is the position vector, $\overset{∙}{q}$ is the speed vector, $x={\left[q^{\left(T\right)},{\overset{∙}{q}}^{\left(T\right)}\right]}^{\left(T\right)}$ is the state vector, *y* is the output vector, *h*(·) is the output function. The control system is supposed equipped by a complete proprioception.

We refer to a generic dynamic system, which may represent both articulated soft robots and biological models (Figures 3A,B), i.e., ẋ(*t*) = *f*(*x*(*t*), *u*(*t*)), *y*(*t*) = *h*(*x*(*t*)), where *f* is the dynamic function, $x={\left[q^{\text{T}},{\overset{∙}{q}}^{\text{T}}\right]}^{\text{T}}\in {\mathbb{R}}^{2n}$ is the state vector, *q* ∈ ℝ^*n*^ are the Lagrangian variables, *y* ∈ ℝ^*l*^ is the output variable, and *h*(*x*) is the output function. It is worth mentioning here that human muscles and agonistic antagonistic variable stiffness actuators share similar characteristics as depicted in Figures 3C,D (Garabini et al., 2017). We propose a bio-mimetic control architecture for bio-inspired robots. The architecture is divided into two layers and summarized in Figure 4. The whole controlled system is organized in four building blocks: the two control levels, the dynamic system, and the output function *h*(*x*) selecting the portion of the state from which depends the task to be accomplished.

The low level features characteristics similar to level A of the Bernstein classification, i.e., it provides low level feedback and dynamic inversion. Thus, it generates as output the efferent action *u* depending on afferent proprioceptive inputs, i.e., $q,\overset{∙}{q}$, and higher level reference ρ ∈ ℝ^*p*^, generated by the high level control, relying on *q* and *y*. Thus, given a desired output trajectory $ŷ:\left[0,t_{\text{f}}\right)\to {\mathbb{R}}^{l}$, where *t*_f_ is the terminal time, the low level control is an appropriate controller able to track that trajectory. On the other hand, the high level control is inspired by level B and level C1 and provides task management.

The low level controller has to present three behaviors: learning by repetition 1, anticipatory behavior 2, and aftereffect over known and unknown trajectories 3. The high level control will present synergistic behavior 4 and ability of re-plan the anticipatory action 5.

To design the control architecture we assume the desired robot impedance behavior as given. Future extension of this work will also consider a direct learning of the optimal impedance depending on the task.

## 4. From Motor Control to Motion Control

In this section we describe the proposed control architecture and its components. To obtain learning by repetition 1 we will employ a learning algorithm able to cope with the non-linear dynamics of the studied class of robots. In particular, we rely on the Iterative Learning Control (ILC) framework (Bristow et al., 2006). The employed ILC method merges a low gain feedback with a feedforward action. Through repetitions the feedforward action will prevail over the feedback action leading to the desired anticipatory behavior 2. It is worth mentioning that ILC is a local method and requires a new learning phase for every novel desired trajectory. Conversely, humans are able to generalize the motion learned through repetitions (Sternad, 2018). To obtain the same feature, we employ Gaussian Process Regression (GPR) (Williams and Rasmussen, 2006) to create a map of learned trajectories. We aim at obtaining also aftereffect, i.e., behavior 3—to test the level of bio-mimecity of the proposed architecture. We base the high level controller on an optimization problem to define the desired task and to solve the redundancy issue. From this optimization problem a synergistic behavior 4 results. Finally, to re-plan an anticipatory action 5 we propose two different approaches, one based on proportional control and the other one based on Model Predictive Control (MPC). Both methods will be tested and compared. We also focus on a trade off between problem dimensionality and accuracy.

### 4.1. Low Level Control

Let us define the error signal as $e:=\hat{x}-x$, where *x* is the measured state vector, while $\hat{x}$ is the desired evolution, given by higher levels of the architecture. In addition, let us define the inverse functional $W:C^{1}\left[0,t_{\text{f}}\right)\to C^{0}\left[0,t_{\text{f}}\right)$, mapping a desired state trajectory $\hat{x}$ into the input û able to track that trajectory. The purpose of the low level controller is to perform dynamic inversion of the system given any desired trajectory $\hat{x}$, thus to find a map approximating *W*. In addition, we aim at replicating the CNS features 1, 2 and 3. To this end, we propose a new algorithm combining Iterative Learning Control (ILC) and Gaussian Process Regression (GPR).

#### 4.1.1. Learning to Track a Trajectory

The learning by repetition behavior 1 can be achieved using a learning technique. Emken et al. (2007) presents a model of learning by repetition process, derived from a statistic model of error evolution over iterations

(1) $$\begin{array}{r} u_{i+1}=\alpha u_{i}+\beta e_{i}, \end{array}$$

where α, β ∈ ℝ^+^ are two positive constants, while *u*_*i*_ and *e*_*i*_ are the control action and the error at the *i*-th iteration, respectively. In this way an input sequence is iteratively computed such that the output of the system is as close as possible to the desired output. Iterative Learning Control (ILC) (Bristow et al., 2006) permits to embed this rule in a general theory, and already achieved good results when applied to VSA robots (Angelini et al., 2018). ILC exploits the whole previous iteration error evolution to update a feedforward command, according to the law

(2) $$\begin{array}{r} u_{i+1}=L\left(u_{i}\right)+z\left(e_{i}\right), \end{array}$$

where the function *z*(*e*_*i*_) identifies the iterative update, while *L*(*u*_*i*_) is a function^1^ mapping the control action of the previous iteration *u*_*i*_ into the current one.

While in works, such as Tseng et al. (2007) is described the pure contribution of error signals, there are evidence, such as Kawato (1996), that feedback motor correction plays a crucial role in motor learning. Hence, a more general algorithm able to merge all of these contribution is needed. Thanks to the described inclusion we can design an ILC controller merging both feedback and feedforward, applying a control law, such as

(3) $$\begin{array}{r} u_{i+1}=L\left(u_{i}\right)+z\left(e_{i},e_{i+1}\right), \end{array}$$

where the presence of the error of the current iteration *e*_*i*+1_ leads to the feedback action. The combination of feedback and feedforward actions, allows to profitably collect sensory-motor memory implementing also the described anticipatory behavior 2. Furthermore, relying mostly on a feedforward action, ILC allows a limited stiffening of the robot (Della Santina et al., 2017a).

Among all the ILC algorithms, in order to opportunely generalize (1) maintaining its intrinsic model-free structure, in this work we use an PD-ILC law in the form of the ones proposed (e.g., in Shou et al., 2003; Ruan et al., 2007), to obtain a minimal dependence on a model of the system dynamics. The proposed approach has been already preliminarily introduced in Angelini et al. (2020a). The adopted iterative update is

(4) $$\begin{array}{r} z\left(t,i\right)=\Gamma_{\text{FFp}}e_{i}\left(t\right)+\Gamma_{\text{FFd}}{ė}_{i}\left(t\right)+\Gamma_{\text{FBp}}e_{i+1}\left(t\right)+\Gamma_{\text{FBd}}{ė}_{i+1}\left(t\right), \end{array}$$

where, *e*_*i*_ is the error evolution at the *i*-th iteration, $\Gamma_{\text{FFp}}\in {\mathbb{R}}^{m\times 2n}$ and $\Gamma_{\text{FFd}}\in {\mathbb{R}}^{m\times 2n}$ are the PD control gains of the iterative update while $\Gamma_{\text{FBp}}\in {\mathbb{R}}^{m\times 2n}$ and $\Gamma_{\text{FBd}}\in {\mathbb{R}}^{m\times 2n}$ are the PD feedback gains. We choose a decentralized structure for the ILC controller, hence, the gain matrices are block diagonal. The gains of the control algorithm can be chosen through several methods. Trial and error approaches could be adopted, but they are usually time consuming and the final performance depends on the experience of the human operator. The ILC framework proposes several techniques to guarantee the convergence of the iterative process depending on the control gains. Thus, other tuning approaches rely on these convergence condition to choose the gains. Some relevant examples of convergence conditions can be found in Arimoto et al. (1984), Ahn et al. (1993), Moore (1999), Bristow et al. (2006), and Wang et al. (2009). In Angelini et al. (2018) an algorithm to automatically tune the control gains is proposed. Finally, it is worth mentioning that the feedback gains should be set low to avoid alteration of the softness of the controlled system (Della Santina et al., 2017a; Angelini et al., 2018).

The adopted solution achieves aftereffect over known trajectories 3. Indeed, the method is able to compensate also unmodeled potential external force field, because it is model-free and learning based. This means that the learned action depends on the external force disturbances that were present during the learning phase. Furthermore, since the method is mostly feedforward, when the external force field is removed, the system presents the desired aftereffect 3.

#### 4.1.2. Generalization of the Learned Trajectories

Given a desired trajectory $\hat{x}$, ILC returns an input û such that $û=W\left(\hat{x}\right)$, thus it returns a pair ($\hat{x}$,$W\left(\hat{x}\right)$). However, the method lacks of generality. Indeed, ILC is a local method, and it requires a novel learning phase for each novel desired trajectory $\hat{x}$. Conversely, humans are capable of effectively performing novel tasks exploiting and generalizing the previously acquired experiences (Sternad, 2018). Angelini et al. (2020b) proposes a method to generalize the control actions w.r.t. to time execution given a limited set of pairs ($\hat{x}$,$W\left(\hat{x}\right)$). Given a desired trajectory $\hat{x}$, the method allows to track $\hat{x}$ with any desired velocity, without any knowledge of the robot model. In this paper, we are interested in generalizing the learning control action w.r.t. the joint evolution, replicating the feature of human beings. To this end, we apply GPR on a set of learned pairs $\left(\hat{x},W\left(\hat{x}\right)\right)$, in order to regress a map—approximating *W*—able to track any novel desired trajectory $\hat{x}$. Then, the system will present also the desired behavior aftereffect over unknown trajectories 3. This is achieved because the regressed map will be based on the learned feedforward control actions.

Several approaches can be applied to compute the inverse functional *W*. Some methods contemplate the independent estimation of a complete model of the system (e.g., Arif et al., 2001; Purwin and D'Andrea, 2009). The limitations of complete model estimation (Nguyen-Tuong et al., 2008) approaches are well-known (e.g., computational onerous). Conversely, in our approach we will focus on a reduced space of control actions and trajectories, in order to limit the computational burden.

*W* is the functional mapping the functional space of the state trajectories into the functional space of the input signals. Computing the regressor of a functional is not a trivial task. For this reason, we reduce the problem complexity limiting our analysis to an approximated solution. In particular we transform the functional *W* into a function through the introduction of two parameterization functions. Then, we focus on the regressor of this approximated solution.

Let us define:

- a parameterization *B* of a subspace of the trajectories space $F\subseteq C^{1}\left[0,t_{\text{f}}\right)$, with dimension *p*, *B*:ℝ^*p*^ → *F*.
- a parameterization *S* of a subspace of the input space $V\subseteq C^{0}\left[0,t_{\text{f}}\right)$, with dimension *d*, *S*:ℝ^*d*^ → *V*.

The trajectory parameterization *B* constraints low level controller to manage only a sub-set *F* of the possible evolutions. The parameterization *S* defines an approximation of control actions, reducing them to the ones included in *V*. Hence, with an abuse of notation, we indicate with *S*^−1^ the application that, given a control action *u*, returns the set of parameters that identifies its approximation, and such that *S*^−1^(*S*(μ)) = μ ∀μ ∈ ℝ^*d*^. Hence *M*(ρ):ℝ^*p*^ → ℝ^*d*^ is so defined

(5) $$\begin{array}{r} M\left(\rho \right):\rho \mapsto S^{-1}\left(W\left(B\left(\rho \right)\right)\right). \end{array}$$

**Figure 5:**
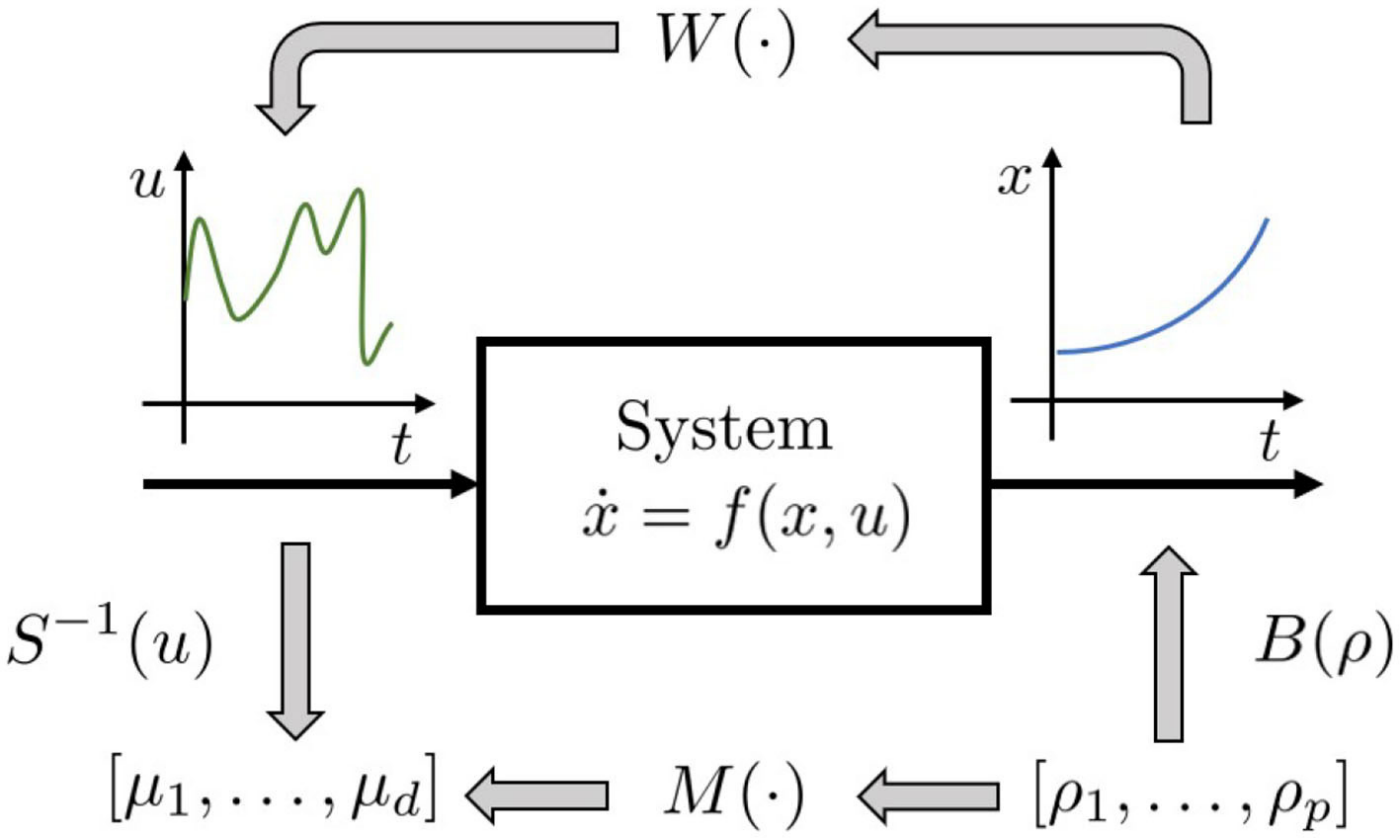
Proposed regression approach: instead of trying to regress the whole inverse functional *W*(·), the idea is to regress the function *M*(·), which provides an approximation [defined by *S*(·)] of control action needed to induce a reduced set of evolution [defined by *B*(·)].

*M*(·) is the map we are interested for (Figure 5). ρ is the array of parameters defining the desired trajectory. The map can then be approximated using a non-linear regression technique. We can then use the approximated map to estimate the control action needed to track a new trajectory. We employ here *Gaussian Process Regression* (GPR), because it achieves good performance, while maintaining low the computational cost. In particular, in the GPR algorithm implementation, we employ the squared exponential as covariance function (Williams and Rasmussen, 2006) described as $k_{\text{c}}\left(x_{1},x_{2}\right)=\sigma_{\text{f}}^{2}e^{\frac{-{\left(x_{1}-x_{2}\right)}^{2}}{2\gamma^{2}}}+\sigma_{\text{n}}\delta \left(x_{1}-x_{2}\right),$where δ(·) is the Kronecker delta, and σ_f_, σ_n_, and γ are free parameters.

Each novel control action will update the map used for generalization. However, to further limit the number of regressed points, for each pair $\left(\bar{\rho },S^{-1}\left(W\left(B\left(\bar{\rho }\right)\right)\right)\right)$, we remove all the stored points from the map which are in a sphere of radius δ_err_, centered in $\bar{\rho }$.

The parametrization of the sub-spaces *F* and *V* can be chosen freely, with the primary goal of keeping low the method complexity without compromising its generality. Several solutions could be implemented and tested. For instance, *F* can be set as a space of polynomial with a fixed order, or as a space of sums of sinusoidal signals. On the other hand, *V* can be approximated as a Gaussian space, or simply a discretization of the signal (Herreros et al., 2016).

Regarding the choice of the sub-space *F*, we would like to adopt trajectories that mimic the human motions. Which are the main characteristics of a motion that make it human-like is still an ongoing debate in literature. In Mombaur et al. (2010), the Authors apply inverse optimal control to define a model of human locomotion path and to exploit it for humanoid robot motion generation. In Tomić et al. (2018) it is studied the problem of human dual-arm motion in presence of contacts with the environment, and it is proposed an algorithm merging inverse optimal control and inverse kinematics to map human motion to humanoid robot motion. An additional method to characterize the human-likeness of robot motion is the adoption of functional synergies directly extracted from human examples as base space (Averta et al., 2017). Without any claim about the solution of this debate, in this work, we adopt the hypothesis formulated in Flash and Hogan (1985) and Friedman and Flash (2009), which states that human movements minimize the jerk. Minimum jerk trajectories are fifth order polynomial (Flash and Hogan, 1985), thus—without any claim of exhaustiveness—we set the vector ρ as the coefficients of the polynomial.

For what concerns the input space parametrization, in this work we focus on piece-wise constant functions with a fixed number *d* of constant length segments, and we implement *S*^−1^ as a time discretization, since it is one of the more natural signal approximation in control. Future work will analyze different choices of parametrization of the input and output spaces.

**Figure 6:**
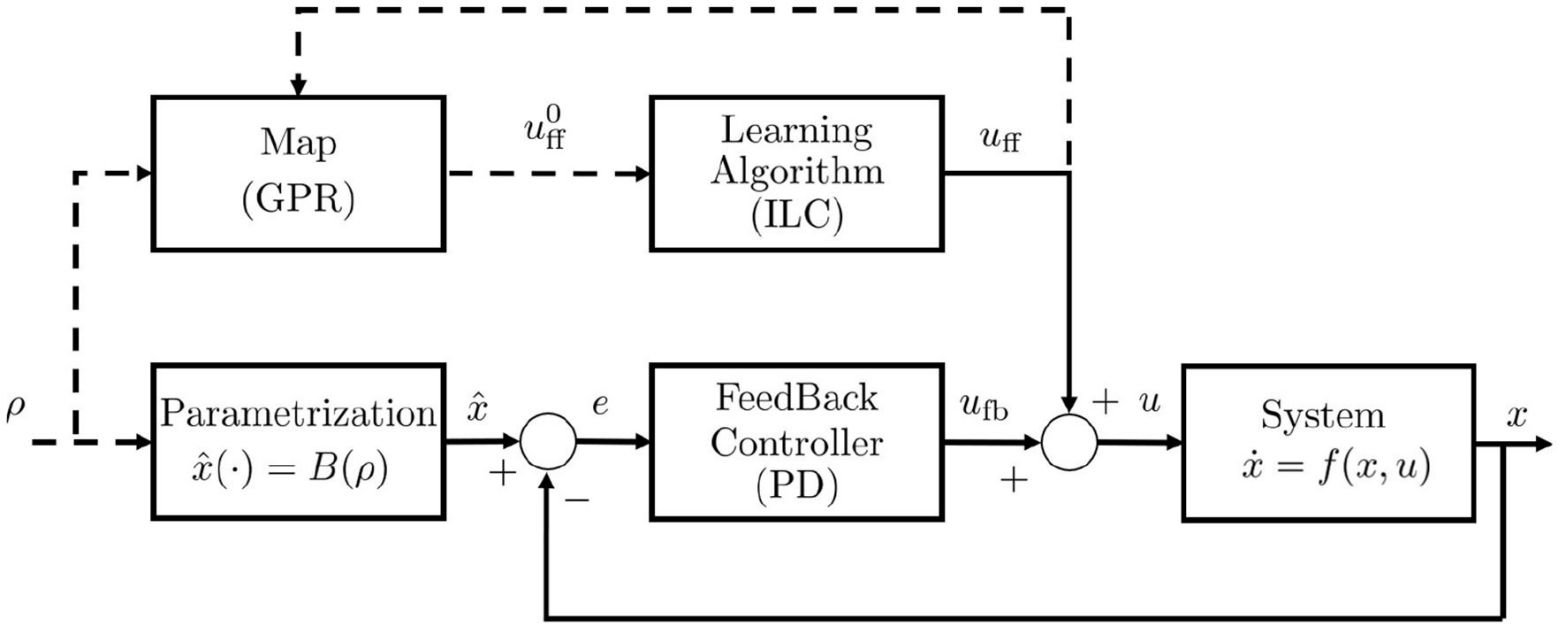
Low level control scheme. *u* = *u*_fb_+*u*_ff_ is the resulting afferent action, and *u*_fb_ and *u*_ff_ are respectively the closed loop and the open loop control components, $u_{\text{ff}}^{0}$ is the a-priori feedforward estimation returned by the map *S*^−1^(*W*(*B*(·))), ρ is the parameter array, *x* is the configuration vector. The *Feedback Controller* is a PD controller, the *Learning Algorithm* is the ILC algorithm, the block *Parametrization* implements the *B*(·) function. Dashed lines indicates flux of information.

In Figure 6 we report the resulting low level control scheme. The input ρ is used in the form of *B*(ρ) as efferent copy for feedback compensation, and through $M\left(\rho \right)=u_{\text{ff}}^{0}$ as estimated anticipatory action. Then, this action can be refined through the learning algorithm. It is worth to be noticed that the proposed low level controller combines learned anticipatory actions and feedback control, working mainly in feedforward when the map reaches the convergence.

It is worth remarking that the adopted solution achieves aftereffect over unknown trajectories 3. Indeed, the regressed map depends on the learned actions. These actions depend on the external force disturbances that were present during the learning phase. Therefore, when the external force field is removed, the system presents the desired aftereffect 3.

The acquired control inputs and, more in general, the regressed map depends on the impedance behavior. This was assumed as provided by an higher level of control in this article (section 3). However, future extension of this work will aim at learning the optimal impedance behavior too, imitating the human capabilities (Burdet et al., 2001). In Mengacci et al. (2020) it is presented a method to decouple the control input to track a trajectory and the control input to regulate the robot impedance, removing the dependency between learned control input and desired stiffness profile. This, in combination with GPR, could be used to generalize the acquired control input w.r.t. the desired stiffness profile and the desired task.

### 4.2. High Level Control

The role of the high level controller is to perform DoFs management in task execution. In particular we are interested in reproducing two of the characteristics of the CNS: synergistic behavior 4 [i.e., given the desired output *h*(*x*), *V*_good_>*V*_bad_ in the configuration space] and re-plan of anticipatory action 5.

The degrees of freedom redundancy in humans is classified as *anatomical*, kinematic or *neurophysiological* (section 2). Here we focus on the kinematic redundancy, and the proposed high level control produces a synergistic behavior for this class of synergies. However, we believe that it could be extended also to the anatomical redundancy. Future work will focus on this point. The neurophysiological redundancy does not have a counterpart in robotics, so it is the Authors' opinion that it is not required to deal with it.

Several works report evidences of the discrete nature of the higher levels of the neural control of movements (e.g., Morasso and Ivaldi, 1982; Loram et al., 2011). In particular, in Neilson et al. (1988) is postulated that the CNS does not plan a new movement until the previous one is finished. This happens because the CNS plan a new motion after receiving the desired perceptual consequences of a movement in a finite interval of time. In order to replicate this behavior we choose a time-discrete control approach. Hereinafter we will use the superscript [*k*], *k* ∈ ℝ to indicate the *k*-th planned movement. Each interval will have the same fixed duration *t*_f_.

Low level controller abstracts the largely unknown and non-linear system into a discrete one which depends on the choice of the subspace. As a trade-off between complexity and accuracy, we heuristically chose a smaller subspace: fifth order monic polynomial with two constraints, which reduces space dimension to 3, while ensuring that subspace elements juxtaposition is of class *C*^2^. In particular we will focus on trajectories fulling these constraints

(6) $$\begin{array}{r} \begin{array}{c} \frac{\partial^{2}q}{\partial t^{2}}{|}_{t=\left\{0,t_{\text{f}}\right\}}=0,\;q_{\text{f}}=q_{\text{s}}+{\overset{∙}{q}}_{\text{f}}t_{\text{f}}, \end{array} \end{array}$$

where *q*_s_ and *q*_f_ are the starting and final values of the polynomials, respectively. Following this choice, we find that $\rho =\left[q_{\text{s}},{\overset{∙}{q}}_{\text{s}},{\overset{∙}{q}}_{\text{f}}\right]$. Given this definition of ρ, the resulting curve is a polynomial spline, and the abstracted dynamics is a discrete integrator

(7) $$\begin{array}{r} q^{\left[k+1\right]}=q^{\left[k\right]}+t_{\text{f}}\rho_{3}^{\left[k\right]}, \end{array}$$

where $\rho_{3}^{\left[k\right]}$ is the third element of ρ^[*k*]^. Note that $\rho_{1}^{\left[k\right]}$ and $\rho_{2}^{\left[k\right]}$ are constrained by the initial conditions, thus they do not appear in (7).

Hence, the high level controller uses ρ as control variable, and its role is to choose the sequence of $\rho_{3}^{\left[k\right]}$, generating a polynomial spline reference.

Level C2 in Bernstein classification (Bemstein, 1967) specifies the task to be accomplished. Analogously, we aim at replicating the same behavior in the proposed high level controller. We define as task a cost function and a set of constraints. Thus, the high level controller is defined by a solver and an optimization problem formulated as

(8) $$\begin{array}{r} \begin{array}{c} \underset{\Delta \rho ,q}{\text{min}}J\left(ŷ-h\left(q\right),q^{\left[k\right]},\Delta \rho_{3}\right)\\ \begin{array}{ccc} \text{s.t.} & \|g_{\text{q}}\left(q^{\left[k\right]}\right)\|\leq \lambda_{\text{q}},\forall k & \\ & \|g_{\rho }\left(\Delta \rho_{3}\right)\|\leq \lambda_{\rho }\\ & q^{\left[k+1\right]}=q^{\left[k\right]}+t_{\text{f}}\rho_{3}^{\left[k\right]}, \end{array} \end{array} \end{array}$$

where *J* is the cost function. *h*(·) is the output function selecting the variables of interest for the task. Δρ_3_ is the difference between two consecutive control commands, i.e., at the *k*-th interval we have $\Delta \rho_{3}:=\rho_{3}^{\left[k\right]}-\rho_{3}^{\left[k-1\right]}$. *g*_q_ and *g*_ρ_ are generic constraint functions, while λ_q_ ∈ ℝ and λ_ρ_ ∈ ℝ are the values of the upper bounds. It is worth noting that ||Δρ_3_||_*R*_ assumes the role of actuation cost, while the difference between the desired and the actual output ||ŷ−*h*(*q*)||_*Q*_ is a metric for performance.

We test two different solvers for the high level control:

- Proportional Control (P): it consists in pre-solving the problem and controlling the system over *x*_opt_ through a proportional controller, which is a dead beat controller for the discrete integrator if $P=t_{\text{f}}^{-1}I$, with the identity matrix.
- Model Predictive Control (MPC): it consists in recalculating the optimum on-line at each time interval, using the first element of the resulting control sequence (Köhler et al., 2020). Conventionally, MPC is hardly applicable to mechanical systems due to their high bandwidths, but the architecture here presented allows MPC application because it is sufficient to apply it only each *t*_f_ seconds.

P control and MPC usually present much different performance and implementation complexity. For this reason, we decided to test both of them to check if a simpler P solver is effective enough, or if the difference in performances can justify the use of a more demanding method, such as MPC.

The high level feedback loop consists in a periodical re-plan of the control sequence, if the actual sensory outcomes are different from the expected ones.

To obtain the desired synergistic behavior 4, we rely on the uncontrolled manifold theory (Scholz and Schöner, 1999). As briefly described in section 2.2, the uncontrolled manifold is the variance through the directions where output is constant and the constraints are verified. This means that the uncontrolled manifold can be identified as the manifold such that *h*(*q*)−ŷ = 0. Focusing on the regulation of the output, rather than on the joint error, is sufficient to obtain the desired synergistic behavior 4.

It is worth noting that the quality of the task execution is strongly affected by the accuracy of the learned low level map. A pre-learning of the map is time consuming and generally not required. So, we will use an online approach to generate the map: if a new task is not properly executed (i.e., its error is greater than a certain threshold η_th_) then the accuracy of the map should be improved through the introduction of a new point, obtained through an ILC execution along the failed trajectory. This approach results in a task-oriented learned map: most of the points will be collected in the portions of the subspace *F* that are more useful for the tasks, obtaining a very good trade-off between map dimension and accuracy.

## 5. Validation

**Figure 7:**
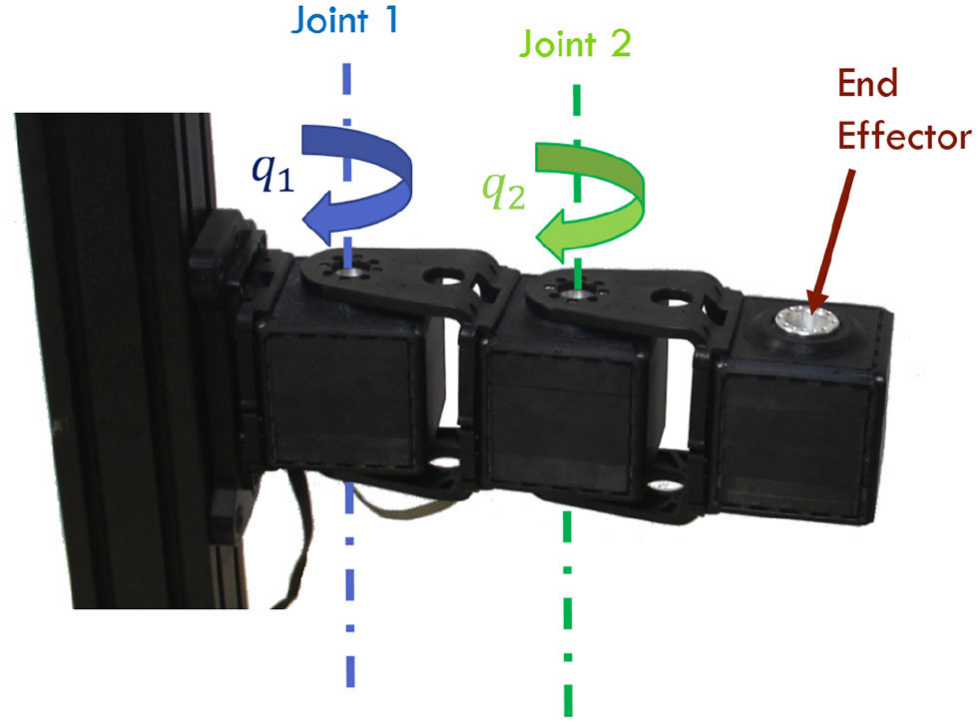
Two degrees of freedom robotic arm used as validation setup. The manipulator is actuated by two *qbmoves Maker Pro*, which are bio-metitic VSAs.

In this section, we test the effectiveness of the proposed control architecture through simulations and experiments. In both cases, we employ as testbed a two degrees of freedom robotic arm, actuated by VSAs (Figure 7). Specifically, we employ two *qbmoves Maker Pro* (Della Santina et al., 2017b), which are bio-metitic variable stiffness actuators presenting characteristics similar to human muscles (Garabini et al., 2017). In both validations we consider the following gains for the algorithm Γ_FFp_ is blkdiag([1, 0.1], [1.25, 0.0375]), Γ_FFd_ is blkdiag([0.1, 0.001], [0.0375, 0.001]), Γ_FBp_ is blkdiag([0.25, 0.025], [0.25, 0.025]), and Γ_FBd_ is blkdiag([0.025, 0.001], [0.025, 0.001]). The parameters of the squared exponential as covariance function in GPR algorithm are σ_f_ = 1, σ_n_ = 0.05, γ = 2, and δ_err_ = π/20.

For performance evaluation we use the error norm 1 of the tracking error evolution, i.e., the integral over time of the norm of the error, *mean error* hereinafter. Furthermore, we refer as *total error* evolution the sum of the absolute tracking error of each joint at a given time.

In section 5.1 we present simulations proving that the proposed control architecture presents the desired behaviors (i)–(v) separately. In section 5.2 we present experiments testing the complete control architecture.

### 5.1. Simulation Results

The employed model is a two degrees of freedom arm. Each link wights 0.5kg and is 0.5m long. Viscous friction equal to 1.2Ns on output shaft is considered. Joints limits are $\left[0,\frac{\pi }{2}\right]\text{rad}$. The model of the actuators takes into account hardware parameters, such as measure noise, communication delays, saturations, motors dynamics^2^. In the following the test separately the low level and the high level controllers.

#### 5.1.1. Low Level Control

In this section, we verify that the proposed low level control achieves the human-like behaviors described in (i)–(iii). We present a set of three simulations to test each behavior. First, we validate the presence of learning by repetition 1 and anticipatory action 2. Then, we test the effectiveness of the learned map. Finally, we verify that the system presents aftereffect over know and unknown trajectories 3.

**Figure 8:**
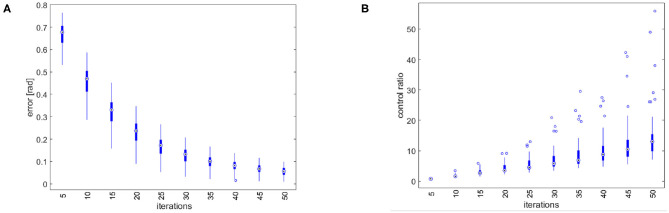
Simulation results of the tracking performance of 50 trajectories randomly selected from *F*. **(A)** Total error over iterations. The control architecture presents the learning by repetitions behavior. **(B)** Ratio between the feedforward and the feedback action. The control architecture presents the anticipatory behavior.

First, we perform trajectory tracking over 50 trajectories randomly selected in *F* through a uniform distribution. Results are shown in Figure 8. Figure 8A shows that the system profitably implements learning by repetition [behavior 1], reducing the error by repeating the same movement. Figure 8B shows that the controller is able to capitalize the sensory-motor memory over a trajectory increasing the role of anticipatory action [behavior 2].

**Figure 9:**
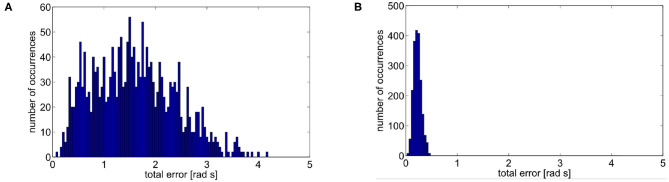
Mean error of 2 · 10^3^ simulations. **(A)** No map is used. The mean error is 1.5929rads with a variance of 0.6272rad^2^s^2^. **(B)** A learned map is used. The mean error is 0.226rads with a variance of 0.0055rad^2^s^2^.

Then, we validate the effectiveness of the map. To this end, we test two scenarios: trajectory tracking without any map and trajectory tracking with a pre-trained map. In the latter case the map is trained on the 50 learning phases performed in the previous simulation. Given the two scenarios, we simulate 2 · 10^3^ trajectories randomly selected in *F* through a uniform distribution. The results are reported in Figure 9. Results show that the performance using the map learned with only 50 random repetitions are more than one order of magnitude better than the ones without the map, and with a sensibly lower variance.

**Figure 10:**
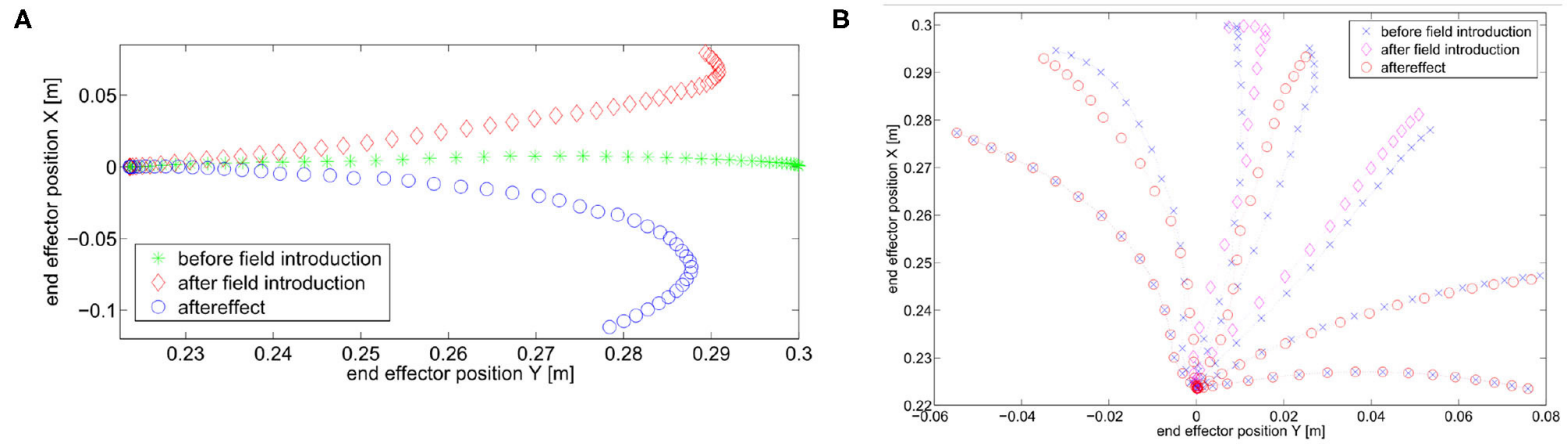
Simulations present aftereffect over known and unknown trajectories. *Before field introduction* are the tracking performance before the introduction of the external force field. The reference trajectory can be considered overlapped. *After field introduction* is the trajectory deformed by the external force field. *Aftereffect* is the trajectory after the field removal. **(A)** Known trajectory. **(B)** Two known trajectories and five unknown trajectories.

Finally, we verify the presence of the aftereffect, i.e., behavior 3. Results are shown in Figure 10, specifically we show aftereffect over known trajectories in Figure 10A, and aftereffect over unknown trajectories in Figure 10B. In the first case, the green asterisk line represents the motion of the robot at the end of the learning phase. Then, we introduce an external force field, which acts on the joints as an external torque described by $\Delta_{1}\left(q,\overset{∙}{q}\right)=-{\overset{∙}{q}}_{1}^{3}-2q_{1}+\pi$ and $\Delta_{2}\left(q,\overset{∙}{q}\right)=-{\overset{∙}{q}}_{2}^{3}-0.4q_{2}$, for the first and second joint, respectively. The trajectory is deformed as a consequence of the force field introduction (diamond red line). We repeat the learning process to recover from performance loss, and the system is again able to follow the initial trajectory (again, green asterisk line). Finally, the field is removed, and the end-effector presents the mirror-image aftereffect, i.e., the trajectory (circle blue line) is specular to the red one.

In the second case we test presence of the aftereffect on unknown trajectories. To this end, we simulate a motor control experiment accounted in Gandolfo et al. (1996). The controller experiences the unknown force field only on two trajectories. In this simulation the external torque is described by $\Delta_{1}\left(q,\overset{∙}{q}\right)=-0.5{\overset{∙}{q}}_{1}-0.15$ and $\Delta_{2}\left(q,\overset{∙}{q}\right)=-0.5{\overset{∙}{q}}_{2}+0.15$. After field removal, we track five additional trajectories. Each one presents aftereffect. Moreover, its effect is more evident near in the trajectories close to the experienced ones. This result proves that the proposed control architecture presents a typical behavior of the CNS, validating its human resemblance.

#### 5.1.2. High Level

In this section, we verify that the proposed high level control achieves the human-like behaviors described in (iv)–(v). We present a set of two simulations to test each behavior. First, we validate the ability to re-plan an anticipatory action 5 and we compare the two approaches (P and MPC). Then, we verify that the system presents a synergistic behavior 4.

**Figure 11:**
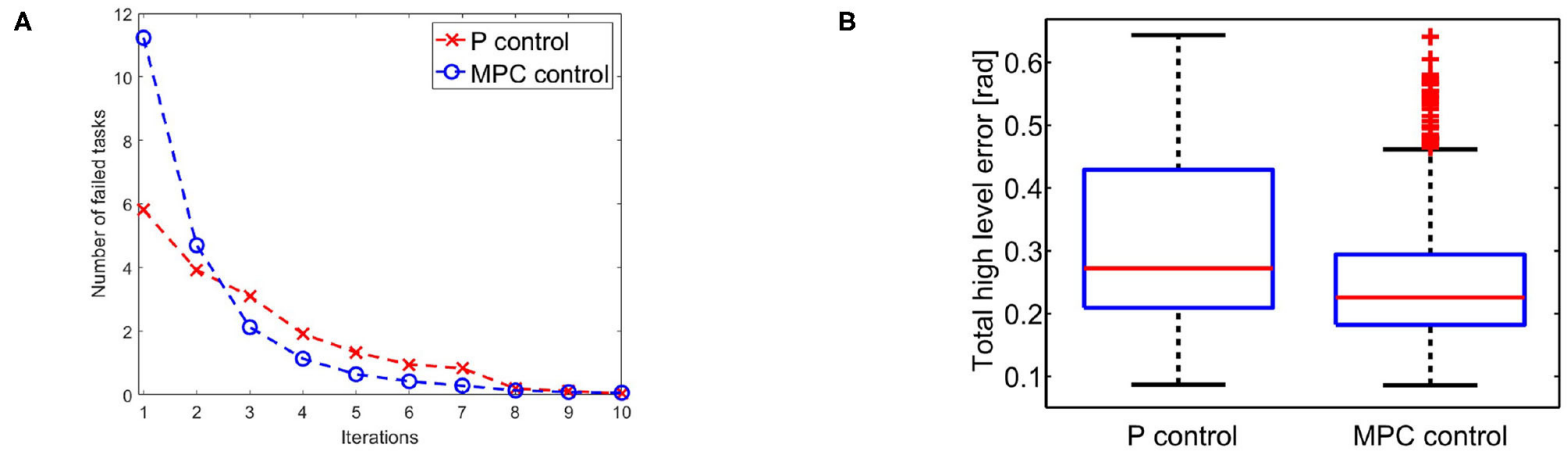
**(A)** Average number of low level evolution tracking which fails the error test at each iteration. **(B)** Error distributions with the two approaches at the first step of the learning process: the MPC approach presents lower error than P approach exploiting the task redundancy.

We evaluate the iterative procedure through 20 tasks. As output we employ the task position of the end-effector along the *x* axis, i.e., *h*(*x*) = *a* cos(*q*_1_) + *a* cos(*q*_1_ + *q*_2_), where *a* is the length of both links. Each task consists in moving the arm such that ||*h*(*x*)−ȳ_*j*_|| is minimized, where ȳ_*j*_ is the desired evolution of task j. The map is regressed online with a threshold $\eta_{\text{th}}=t_{\text{f}}\frac{\pi }{10}=\frac{\pi }{20}$. This means that there is no pre-learned map and a new learning process is executed each time the tracking error is greater than η_th_. Figure 11 shows the result. Figure 11A reports the average number of sub-tasks that presents error greater than η_th_ at each iteration. It is worth noting that the map converges to a complete representation of the inverse system, i.e., no more learning is needed, after ~8 tasks, with both P and MPC algorithms. Figure 11B shows that the MPC performance are better than the P one. This occurs thanks to the re-optimization at each iteration that permits to fully exploit task redundancies. In other terms, if the system moves to a state $\tilde{x}$ different from the desired one $\hat{x}$, but such that $h\left(\tilde{x}\right)=h\left(\hat{x}\right)$, then the P controller reacts trying to regulate the two states to be the same, while the MPC recognizes that the task is accomplished and does not generate any further control action.

**Figure 12:**
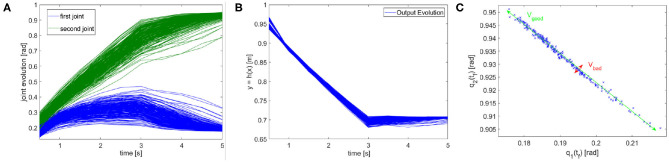
Synergistic behavior. The same task is executed 250 times with randomly selected initial conditions using a normal distribution with standard deviation equal to 0.03 and mean value equal to the correct initial condition value. **(A)** The evolution of the joint present high variability. **(B)** The evolution in the task space presents an analogous behavior, thus the performance are unvaried. **(C)** The distribution in configuration space highlights the synergy-like behavior of the high level controller.

In terms of tracking, the P controller presents good performance but worse than MPC. Therefore, due to the greater complexity of the latter method it would be possible to opt for the P controller. However, we are also interested in obtaining a synergistic behavior 4. To this end, the MPC approach is preferable. To verify the presence of the synergistic behavior 4, we track a reference trajectory with different initial conditions. In particular, we randomly select 250 initial conditions using a normal distribution with standard deviation equal to 0.03 and mean value equal to the correct initial condition value. Figure 12A shows high variability in joints evolution, while Figure 12B highlights that the task performance are preserved. Considering the definition of synergy reported in section 4.1, this simulation shows the presence of a synergistic behavior of the controlled system, presenting *V*_good_ >> *V*_bad_ in the configuration space (Figure 12C).

### 5.2. Experimental Results

In this section we test the complete control architecture, and we verify that it presents the desired behavior (i)–(v). Three experiments are presented, one testing the learning by repetition 1 and anticipatory behavior 2, one testing the aftereffect 3, and one testing the performance of the online map learning. It is worth noting that the reference trajectory is provided by the high level control, validating the complete architecture.

The robotic platform is the two degrees of freedom planar arm depicted in Figure 7. The output function *h*(*x*) is the end-effector position given by *h*(*x*) = [*b* cos(*q*_1_) + *b* cos(*q*_1_ + *q*_2_), *b* sin(*q*_1_) + *b* sin(*q*_1_ + *q*_2_)], where *b* = 0.1m is the length of the links. Given a desired position ȳ, and a discrete time interval $\bar{k}$, the experimental task is to maximize the velocity of the end effector in the desired position ȳ at the desired time step $\bar{k}$. This task can be modeled as the optimization problem

(9) $$\begin{array}{l} \underset{\Delta \pi ,q}{\min}\|\bar{y}-h(q^{\left[\bar{k}\right]}){\|}_{Q_{\text{p}}}-\|h(q^{\left[\bar{k}\right]})-h(q^{\left[\bar{k}-1\right]}){\|}_{Q_{\text{v}}}+\|\Delta \pi {\|}_{R}\\ \text{s.t. }{\underline{\lambda }}_{\text{q}}\geq q^{\left[k\right]}\geq {\bar{\lambda }}_{\text{q}}\;,\;\forall k=1,\ldots ,10\\ \;q^{\left[k+1\right]}=q^{\left[k\right]}+t_{\text{f}}\pi_{3}^{\left[k\right]}\;,\;\forall k=1,\ldots ,9, \end{array}$$

where ${\underline{\lambda }}_{\text{q}}$ and ${\bar{\lambda }}_{\text{q}}$ are the joint limits. *R*, *Q*_p_ and *Q*_v_ are the weight matrices of the input, the final position cost, and the final velocity, respectively, and their value is set as *R* = 0.1*I*_20×20_, *Q*_p_ = 20*I*_2×2_, and *Q*_v_ = 10*I*_2×2_.

**Figure 13:**
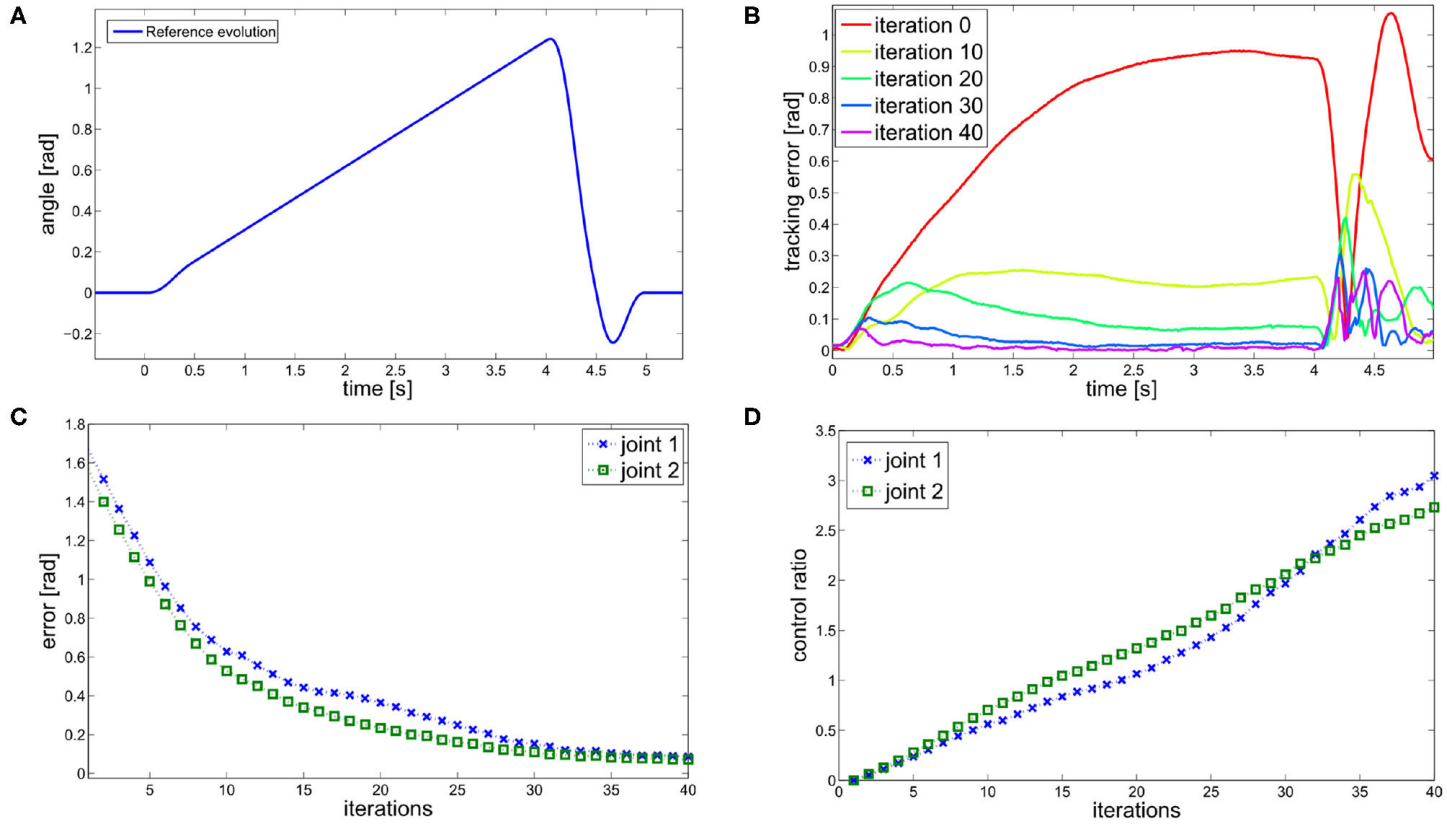
ILC experiment. **(A)** Reference trajectory resulting from the optimization problem (9). The trajectory is equal for both joints. **(B)** Tracking error evolution for different meaningful iteration of the ILC algorithm. **(C)** The evolution of the error over iterations shows the learning by repetitions behavior. **(D)** The ratio between feedforward and feedback actions shows an anticipatory behavior.

Figure 13A shows the solution of the optimization problem (9) with parameters *t*_f_ = 0.5s, ${\underline{\lambda }}_{\text{q}}={\left[0,0\right]}^{\text{T}}$ and ${\bar{\lambda }}_{\text{q}}={\left[\pi /2,\pi /2\right]}^{\text{T}}$, $\bar{k}=9$, ȳ = [0.20]^T^. This is the reference trajectory of the fist experiment, and it is equal for both joints.

The results are shown in Figure 13. The proposed algorithm learns the task through repetitions: in 40 iterations the achieved performance are satisfying. Figure 13B shows the tracking error evolution over time, for a few meaningful iterations. Figure 13C proves that the system implements learning by repetition [behavior 1], reducing the error exponentially by repeating the same movement. The mean error decreases approximately about 63.7% w.r.t. its initial value in 10 iterations, and of the 95% in 40 iterations. Finally, Figure 13D depicts the ratio between total feedforward and feedback action, over learning iterations. This shows the predominance of anticipatory action at the growth of sensory-motor memory [behavior 2]. It is worth to be noticed that feedback it is not completely replaced by feedforward, which is coherent with many physiological evidences (e.g., Shadmehr et al., 2010).

**Figure 14:**
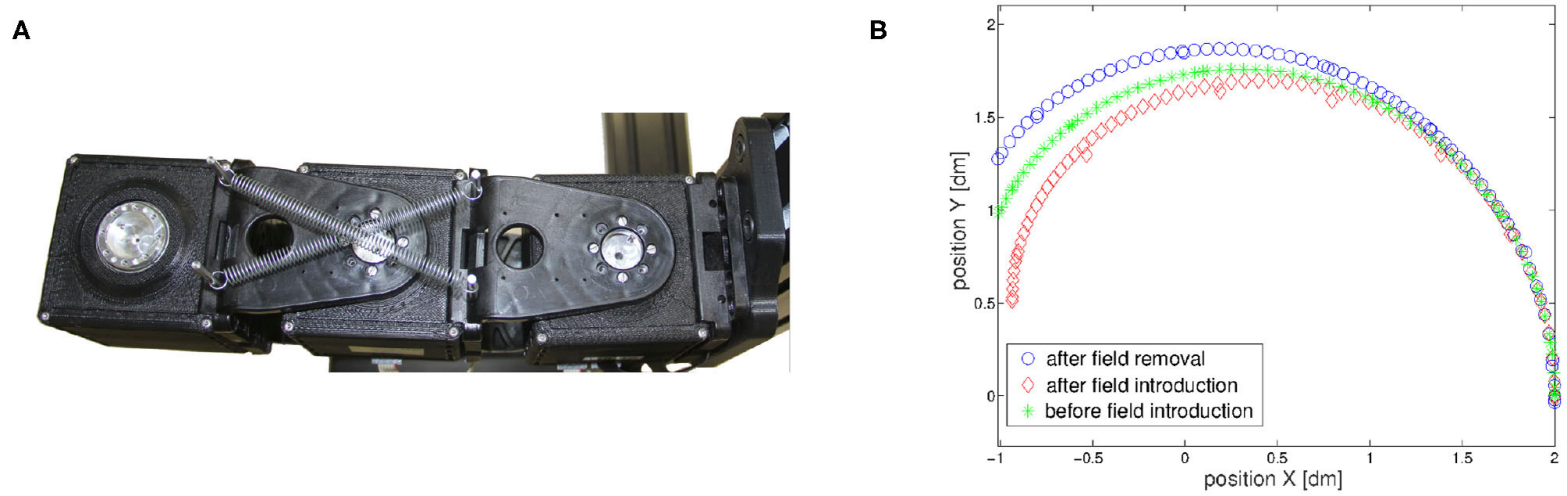
The designed control architecture presents aftereffect on known trajectories. **(A)** An unknown external force field is applied to the robotic arm through the addition of springs. **(B)** The introduction of the force field deforms the trajectory (red line) After some repetitions the strict movement is recovered. If the force field is then removed the trajectory (blue line) is deformed in a way specular to the first deformation.

The second experiment has two goals. First, it tests the ability of the control algorithm to cope with aggressive external disturbances as springs in a parallel configuration (Figure 14A). Then, it validates the presence of mirror-image aftereffect [behavior 3]. The robotic arm learns to move its end-effector following the movement depicted in Figure 14B (green asterisk line). After the learning process we introduced an external force field. The unknown external force field is generated by a couple of springs of elastic constant 0.05Nm^−1^, connected as in Figure 14A. Due to the spring introduction, the robot end-effector evolution is altered as depicted in Figure 14B (red diamond line). At this point, the algorithm recovers the original performance after few iterations, proving its ability to cope with external disturbances (learning process not shown for the sake of clarity). Finally the springs are removed, and the end-effector follows a trajectory (blue circle line in Figure 14B), which is the mirror w.r.t. the nominal one, of the one obtained after field introduction, therefore proving the ability of the proposed algorithm to reproduce mirror-image aftereffect [behavior 3].

To conclude we test the map in the complete control architecture. The idea is to repeatedly perform similar tasks, and to quantify the map performance. In particular, we are interested in verifying that the map capitalizes upon the information of the previous task executions in the new trials. In this experiment, we sequentially perform 10 tasks. The task parameters are *t*_f_ = 0.5s, ${\underline{\lambda }}_{\text{q}}={\left[0,0\right]}^{\text{T}}$ and ${\bar{\lambda }}_{\text{q}}={\left[\pi /2,\pi /2\right]}^{\text{T}}$, and ȳ = [0.20]^T^. In this experiment, $\bar{k}$ is chosen randomly with a uniform distribution in the interval {2, …, 10} for each task. This means that each task aims to maximize the link velocity at a different time step. The resulting trajectory has a form similar to the one depicted in Figure 13A, eventually scaled on the abscissa axis respect to the value of $\bar{k}$, and on the ordinate respect to the values of ${\underline{\lambda }}_{\text{q}}$ and ${\bar{\lambda }}_{\text{q}}$: the system moves as slow as possible (i.e., in $\bar{k}-1$ steps) in the configuration that is most distant from the starting point (i.e., ${\bar{\lambda }}_{\text{q}}$), then in a time step it moves at the maximal possible speed to the initial position, finally it remains stationary.

For each task we performed a learning process lasting for 40 iterations. The resulting low level control is used for map regression. This process is repeated 20 times. Hereinafter each of these repetition is referred as *trial*. To analyze the results we define two error metrics *E* and *I*^*i*^. For every *i*-th task in the *j*-th trial we evaluate (i) $e_{\text{nm}}^{i,j}$, i.e., the tracking error without the use of the map, and (ii) $e_{\text{wm}}^{i,j}$, i.e., the tracking error with the map learned with previous trajectories.

It is worth to be noticed that both error values $e_{\text{nm}}^{i,j}$ and $e_{\text{wm}}^{i,j}$ are not correlated with index *j*. However, while $e_{\text{nm}}^{i,j}$ is neither correlated with index *i*, $e_{\text{wm}}^{i,j}$ appears to be correlated with task *i*, due to the presence of the map.

What we are interested in evaluating is how much the error $e_{\text{wm}}^{i,j}$ decreases respect to the performance without map $e_{\text{nm}}^{i,j}$. Hence we define the metric

(10) $$\begin{array}{r} E=\frac{1}{N_{\text{i}}N_{\text{j}}}\underset{\begin{array}{c} i=1,\ldots ,N_{\text{i}}\\ j=1,\ldots ,N_{\text{j}} \end{array}}{\sum }\left(\frac{1}{T}\overset{T}{\underset{0}{\int }}\|e_{\text{nm}}^{i,j}\left(t\right)\|\text{d}t\right), \end{array}$$

where *T* = 10*t*_f_ is the task duration, *N*_i_ = 10 is the number of tasks in a sequence of learning, *N*_j_ = 20 is the number of trials. Hence *E* is the mean value of error without map, and it will be used for normalization purpose.

Therefore the considered error index for the *i*-th task is defined as

(11) $$\begin{array}{r} I^{i}=\frac{1}{E}\frac{1}{N_{\text{j}}}\underset{j=1,\ldots ,N_{\text{j}}}{\sum }\left(\frac{1}{T}\overset{T}{\underset{0}{\int }}\|e_{\text{wm}}^{i,j}\left(t\right)\|\text{d}t\right). \end{array}$$

*I*^*i*^ represents the normalized mean controlled system behavior over trials at the *i*-th task. *I*^*i*^ > 1 indicates that the map degrades the performance of the system, *I*^*i*^ = 1 indicates that the map does not modify the system behavior, *I*^*i*^ ∈ [0, 1) indicates that the map increases the system performance.

However, it is worth noticing that the regressed map has the goal of improving the performance also of trajectories that differ from the ones stored in the map itself. In particular, the regressed map aims at improving the performance of *dynamically similar* tasks, while maintaining unaltered the performance of *dynamically different* tasks. To analyze this point, we test it in presence of a novel different trajectory *w*. $I_{\text{w}}^{i}$ represent index (11) for the novel reference. Specifically, the employed trajectories are: *s*, i.e., *dynamically similar*, and *r*, i.e., *dynamically different*

(12) $$\begin{array}{r} s_{k}=\frac{\pi }{4}\sin\left(\frac{3\pi }{2}k\right)\left[\begin{array}{c} 1\\ 1 \end{array}\right],\;r_{k}=\frac{\pi }{4}\sin\left(\frac{3\pi }{2}k\right)\left[\begin{array}{c} -2\\ 1 \end{array}\right]. \end{array}$$

**Figure 15:**
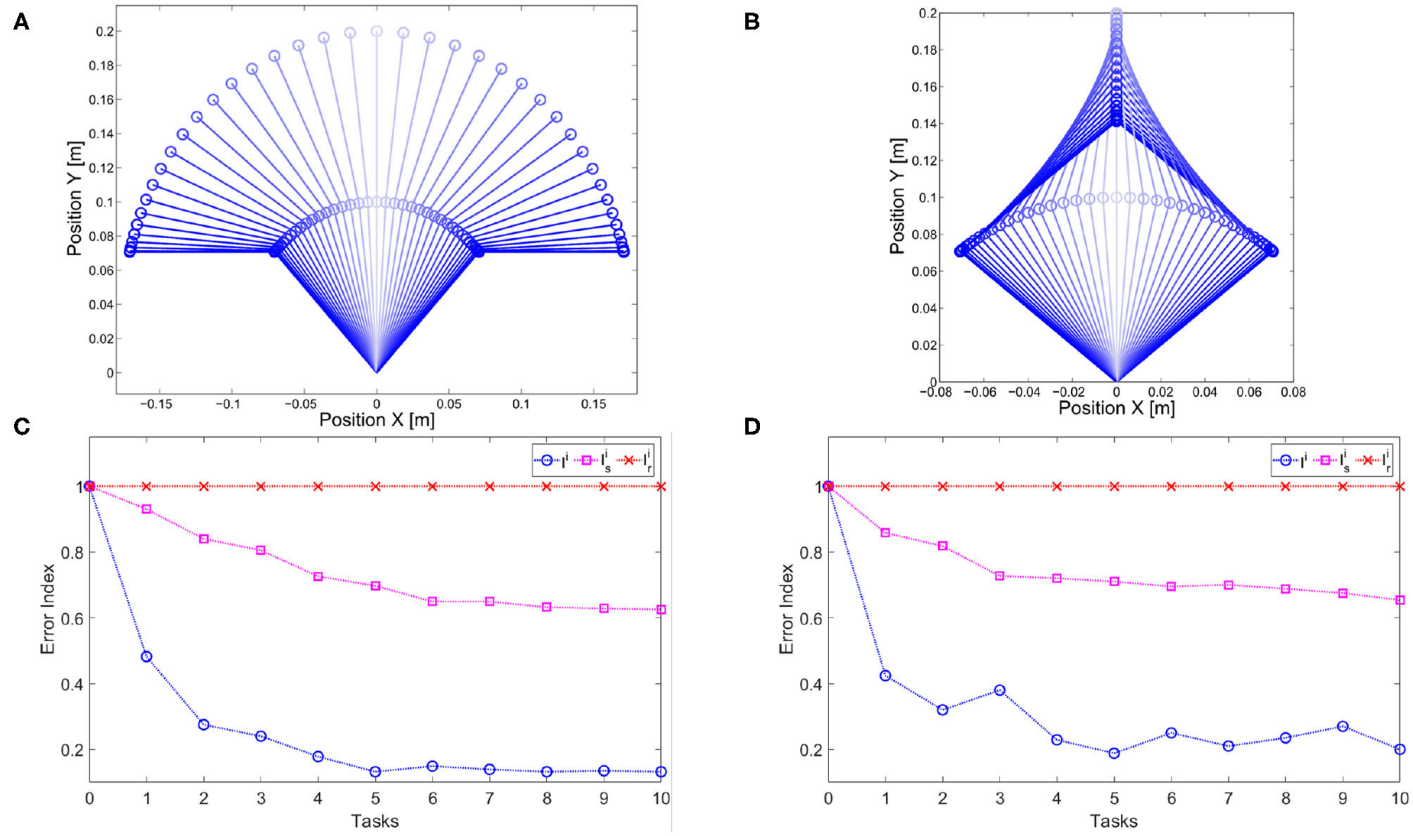
Experimental map evaluation. **(A)** *Dynamically similar* trajectory. **(B)** *Dynamically different* trajectory. Evolution of the error index (11) used for map evaluation in soft **(C)** and stiff **(D)** scenario. The error index *I*^*i*^ on the set of tasks of interest converges to the best reachable performance after ~5 tasks in both cases. Then, two different trajectories are tested: *s* which is *dynamically similar* and *r* which is *dynamically different*. The map reduces the error on the *dynamically similar* trajectory ($I_{\text{s}}^{i}$), and it leaves unadulterated the performance on the *dynamically different* trajectory ($I_{\text{r}}^{i}$).

The two trajectories are presented in Figures 15A,B, respectively. It is worth noticing that the *s* motion is more similar to the task trajectories than the *r* motion since both joint evolution are concordant.

This experiment has been performed with two different scenarios: low and high stiffness. The results are reported in Figures 15C,D, respectively. Both figures show that the map converges to a complete inversion of the system in the set of tasks of interest in ~5 iterations, i.e., when five tasks are included in the map there is no more improvement and the best performance are achieved. Furthermore, the method is able to reduce the error on the trajectory dynamically similar, without degrading the performance of the trajectory dynamically different. This result is achieved both in the low stiffness case and in the high one.

## 6. Conclusions and Future Work

In this work a novel control architecture that simultaneously shows the main characteristics of human motor control system (learning by repetition, anticipatory behavior, aftereffect, synergies) has been stated. The effectiveness of the proposed control framework has been validated in simulations and via experimental tests. The experiments have been conducted on a robotic platform, the qbmoves, closely resembling the muscular system and in which the control inputs, namely reference position and stiffness preset, have their biological counterpart in the reciprocal and co-activation, as per Equilibrium Point Hypothesis. The proposed control architecture translates elements of the main motor control theories in well-stated mechanisms belonging to control theory. Control Engineering could provide a useful framework for theory falsification in motor control, and it could give an already well-formed global language for problem definition. Furthermore, human behavior can be used to ensure human-like performance in robotic systems, and hence be used as a starting point for novel control models. We will further analyze this point in future work.

Future work will also aim at increasing the human-likeness of the proposed control architecture. First we will focus on merging the generalization method proposed in Angelini et al. (2020b) and the generalization method based on GPR that was presented in this paper. The union of the two approaches will grant to the robot the ability to track any desired trajectory, with any desired velocity, considerably limiting the amount of required learning procedures. This solution will further close the gap between robot and human capability in terms of previous experience exploitation. Then, we will aim at replicating the impedance behavior learning that is typical of human beings, and it is generally related to the performed task. Indeed, thanks to our control architecture the robot compliance is not altered, meaning that it can be freely exploited. Additionally, we will exploit functional synergies extracted from recorded human motions to increase the human-likeness of the robot movements (Averta et al., 2020). Finally, this work focused on robot powered by mono-articular actuators, i.e., platforms where each motor separately drives each link. However, some systems, e.g., human musculoskeletal system, present a poly-articular structure. In Mengacci et al. (2020), a few preliminary insights about the application of ILC to poly-articular systems have been discussed. Starting from these results, future work will also study the application of the proposed control architecture to poly-articular robots, achieving also a anatomical synergistic behavior.

## Data Availability Statement

The raw data supporting the conclusions of this article will be made available by the authors, without undue reservation.

## Author Contributions

FA and CD developed the method and equally contributed to the paper. CD performed the experiments. All authors conceived the idea together and contributed to writing the manuscript.

## Conflict of Interest

The authors declare that the research was conducted in the absence of any commercial or financial relationships that could be construed as a potential conflict of interest.

## Footnotes

^1^*L*(*u*_*i*_) is typically a smoothing function.

^2^The simulink model is available online at www.naturalmachinemotioninitiative.com.
